# Genome sequence of *Rhizobium leguminosarum* bv *trifolii* strain WSM1689, the microsymbiont of the one flowered clover *Trifolium uniflorum*

**DOI:** 10.4056/sigs.4988693

**Published:** 2013-12-31

**Authors:** Jason Terpolilli, Tian Rui, Ron Yates, John Howieson, Philip Poole, Christine Munk, Roxanne Tapia, Cliff Han, Victor Markowitz, Reddy Tatiparthi, Konstantinos Mavrommatis, Natalia Ivanova, Amrita Pati, Lynne Goodwin, Tanja Woyke, Nikos Kyrpides, Wayne Reeve

**Affiliations:** 1 Centre for Rhizobium Studies, Murdoch University, Perth, Australia; 2 Department of Agriculture and Food, Western Australia, Australia; 3 Department of Plant Sciences, University of Oxford, UK; 4 Sir Walter Murdoch Adjunct Professor, Murdoch University, Perth, Australia; 5 Los Alamos National Laboratory, Bioscience Division, Los Alamos, New Mexico, USA; 6 Biological Data Management and Technology Center, Lawrence Berkeley National Laboratory, Berkeley, California, USA; 7 DOE Joint Genome Institute, Walnut Creek, California, USA

**Keywords:** root-nodule bacteria, nitrogen fixation, lupin-nodulating, rhizobia, *Alphaproteobacteria*

## Abstract

*Rhizobium leguminosarum* bv. *trifolii* is a soil-inhabiting bacterium that has the capacity to be an effective N_2_-fixing microsymbiont of *Trifolium* (clover) species. *R. leguminosarum* bv. *trifolii* strain WSM1689 is an aerobic, motile, Gram-negative, non-spore-forming rod that was isolated from a root nodule of *Trifolium uniflorum* collected on the edge of a valley 6 km from Eggares on the Greek Island of Naxos. Although WSM1689 is capable of highly effective N_2_-fixation with *T. uniflorum*, it is either unable to nodulate or unable to fix N_2_ with a wide range of both perennial and annual clovers originating from Europe, North America and Africa. WSM1689 therefore possesses a very narrow host range for effective N_2_ fixation and can thus play a valuable role in determining the geographic and phenological barriers to symbiotic performance in the genus *Trifolium.* Here we describe the features of *R. leguminosarum* bv. *trifolii* strain WSM1689, together with the complete genome sequence and its annotation. The 6,903,379 bp genome contains 6,709 protein-coding genes and 89 RNA-only encoding genes. This multipartite genome contains six distinct replicons; a chromosome of size 4,854,518 bp and five plasmids of size 667,306, 518,052, 341,391, 262,704 and 259,408 bp. This rhizobial genome is one of 20 sequenced as part of a DOE Joint Genome Institute 2010 Community Sequencing Program.

## Introduction

The nitrogen (N) cycle is one of the most important biogeochemical processes underpinning the existence of life on Earth. A key step in this cycle is to convert relatively inert atmospheric dinitrogen (N_2_) into a bioaccessible form such as ammonia (NH_3_) through a process referred to as biological nitrogen fixation (BNF). BNF is performed only by a specialized subset of *Bacteria* and *Archaea* that possess the necessary cellular machinery to enzymatically reduce N_2_ into NH_3_. Some of these bacteria (termed rhizobia or root nodule bacteria) have evolved non-obligatory symbiotic relationships with legumes whereby the bacteria receive a carbon source from the plant and in return supply fixed N to the host [1]. Harnessing this association can boost soil N-inputs and therefore production yields of legumes, or non-legumes grown in subsequent years, without the need for supplementation with industrially synthesized N-based fertilizers [2].

Some of the most widely cultivated pasture legumes are members of the legume genus *Trifolium* (clover). The natural distribution of these species spans three centers of diversity, with an estimated 28% of species in the Americas, 57% in Eurasia and 15% in sub-Saharan Africa [3]. Approximately 30 species of clover, predominately of Eurasian origin, are widely grown as annual and perennial species in pasture systems in Mediterranean and temperate climatic zones [3]. Globally-important perennial species of clover include *T. repens* (white clover), *T. pratense* (red clover), *T. fragiferum* (strawberry clover) and *T. hybridum* (alsike clover). While clovers are known to form N_2_-fixing symbiotic associations with *Rhizobium leguminosarum* bv. *trifolii,* there exists wide variation in symbiotic compatibility across different strains and hosts from ineffective (non-N_2_-fixing) nodulation to fully effective N_2_-fixing partnerships.

*Rhizobium leguminosarum* bv. *trifolii* strain WSM1689 was isolated in 1995 from a nodule of the perennial clover *Trifolium uniflorum* collected on the edge of a valley 6 km from Eggares on the Greek Island of Naxos. *T. uniflorum* is one of small number of perennial *Trifolium* spp. found in the dry, Mediterranean basin. While WSM1689 has been shown to be either ineffective or unable to nodulate a range of annual and perennial *Trifolium* sp., it is a highly effective N_2_-fixing microsymbiont of *T. uniflorum* [4]. Therefore, *R. leguminosarum* bv. *trifolii* WSM1689 has a very narrow host range and thus represents a good isolate to study the genetic basis of symbiotic specificity. The availability of this sequence data also complements the already published genomes of the clover-nodulating *R. leguminosarum* bv. *trifolii* WSM1325 [5] and WSM2304 [6]. Here we present a summary classification and a set of general features for *R. leguminosarum* bv. *trifolii* strain WSM1689 together with the description of the complete genome sequence and its annotation.

## Classification and features

**Figure 1:**
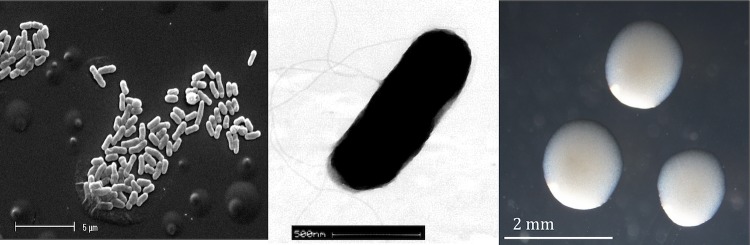
Images of *Rhizobium leguminosarum* bv. *trifolii* strain WSM1689 using scanning (Left) and transmission (Center) electron microscopy and the appearance of colony morphology on ½LA (Right).

*R. leguminosarum* bv. *trifolii* strain WSM1689 is a motile, non-sporulating, non-encapsulated, Gram-negative rod in the order *Rhizobiales* of the class *Alphaproteobacteria*. The rod-shaped form varies in size with dimensions of approximately 0.25-0.5 μm in width and 2.0 μm in length (Figure 1 Left and 1 Center). It is fast growing, forming colonies within 3-4 days when grown on half strength Lupin Agar (½LA) [7], tryptone-yeast extract agar (TY) [8] or a modified yeast-mannitol agar (YMA) [9] at 28°C. Colonies on ½LA are opaque, slightly domed and moderately mucoid with smooth margins (Figure 1 Right). Minimum Information about the Genome Sequence (MIGS) is provided in Table 1.

**Table 1:** Classification and general features of *Rhizobium leguminosarum* bv. *trifolii* strain WSM1689 according to the MIGS recommendations [10,11].

**MIGS ID**	**Property**	**Term**	**Evidence code**
	Current classification	Domain *Bacteria*	TAS [11]
		Phylum *Proteobacteria*	TAS [12]
		Class *Alphaproteobacteria*	TAS [13,14]
		Order *Rhizobiales*	TAS [14,15]
		Family *Rhizobiaceae*	TAS [16,17]
		Genus *Rhizobium*	TAS [16,18-21]
		Species *Rhizobium leguminosarum bv. trifolii*	TAS [16,18,21,22]
		Strain WSM1689	TAS [4]
	Gram stain	Negative	IDA
	Cell shape	Rod	IDA
	Motility	Motile	IDA
	Sporulation	Non-sporulating	NAS
	Temperature range	Mesophile	NAS
	Optimum temperature	28°C	NAS
	Salinity	Not reported	NAS
MIGS-22	Oxygen requirement	Aerobic	TAS [4]
	Carbon source	Varied	NAS
	Energy source	Chemoorganotroph	NAS
MIGS-6	Habitat	Soil, root nodule, host	TAS [4]
MIGS-15	Biotic relationship	Free living, symbiotic	TAS [4]
MIGS-14	Pathogenicity	Non-pathogenic	NAS
	Biosafety level	1	NAS [23]
	Isolation	Root nodule	TAS [4]
MIGS-4	Geographic location	Naxos, Greece	IDA
MIGS-5	Nodule collection date	1995	IDA
MIGS-4.1 MIGS-4.2	Latitude Longitude	37.128333 25.443333	IDA IDA
MIGS-4.3	Depth	Not reported	
MIGS-4.4	Altitude	Not reported	

Evidence codes – IDA: Inferred from Direct Assay; TAS: Traceable Author Statement (i.e., a direct report exists in the literature); NAS: Non-traceable Author Statement (i.e., not directly observed for the living, isolated sample, but based on a generally accepted property for the species, or anecdotal evidence). These evidence codes are from the Gene Ontology project [24].

**Figure 2:**
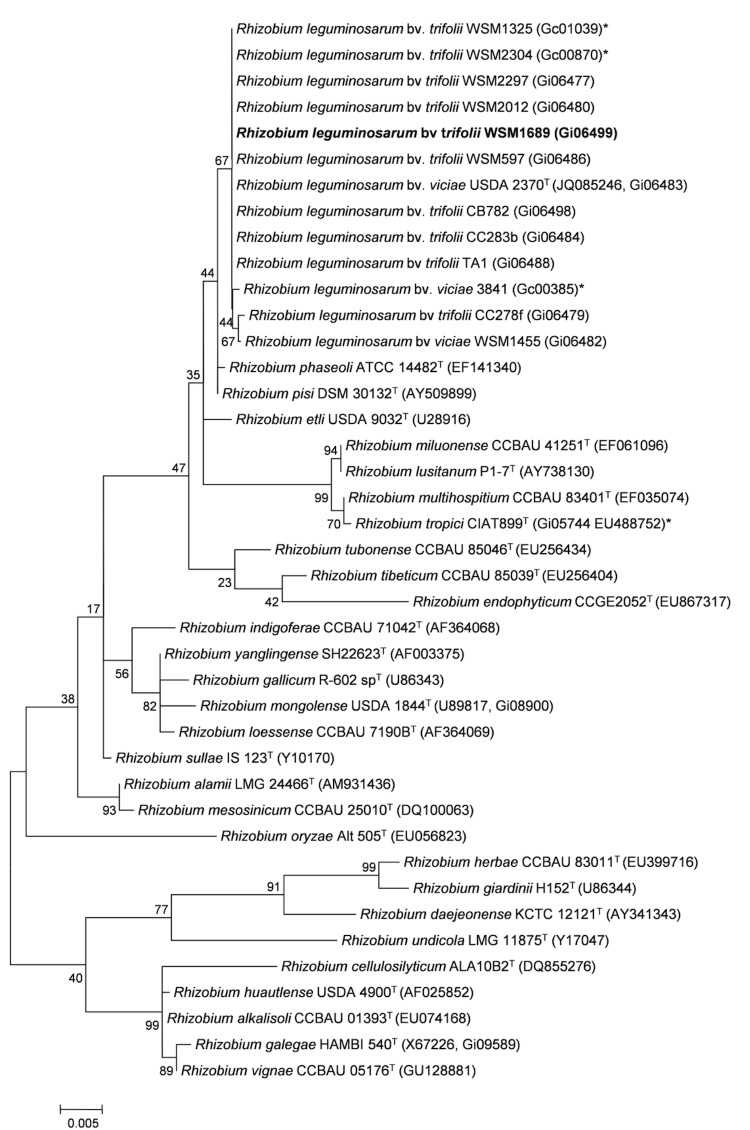
Phylogenetic tree showing the relationship of *Rhizobium leguminosarum* bv *trifolii* WSM1689 (shown in bold print) to other root nodulating *Rhizobium* spp. in the order *Rhizobiales* based on aligned sequences of the 16S rRNA gene (1,180 bp internal region). All positions containing gaps and missing data were eliminated. All sites were informative and there were no gap-containing sites. Phylogenetic analyses were performed using MEGA, version 5 [25]. The tree was built using the Maximum-Likelihood method with the General Time Reversible model [26]. Bootstrap analysis [27] with 500 replicates was performed to assess the support of the clusters. Type strains are indicated with a superscript T. Brackets after the strain name contain a DNA database accession number and/or a GOLD ID (beginning with the prefix G) for a sequencing project registered in GOLD [28]. Published genomes are indicated with an asterisk.

Figure 2 shows the phylogenetic neighborhood of *R. leguminosarum* bv. *trifolii* strain WSM1689 in a 16S rRNA gene sequence based tree. This strain shares 100% (1362/1362 bp) sequence identity to the 16S rRNA gene of *R. leguminosarum* bv. *trifolii* strain WSM1325 [5] and *R. leguminosarum* bv. *trifolii* strain WSM2304 [6].

### Symbiotaxonomy

*R. leguminosarum* bv. *trifolii* WSM1689 is a highly effective microsymbiont of the perennial Eurasian clover *Trifolium uniflorum* (Table 2). In contrast, WSM1689 does not nodulate the perennial *T. fragiferum* and forms white ineffective (Fix^-^) nodules with other perennial and annual clovers of Eurasian origin. Moreover, WSM1689 is either Nod^-^ or Fix^-^ on clovers of North American or African origin. Therefore, WSM1689 is unusual in having an extremely narrow clover host range for the establishment of effective N_2_-fixing symbiosis.

**Table 2:** Compatibility of WSM1689 with both perennial and annual *Trifolium* genotypes for nodulation (Nod) and N_2_-Fixation (Fix). Data compiled from [4].

Species Name	Cultivar	Origin	Growth habit	Nod	Fix	Comment
*T. uniflorum*	Nil	Europe	Perennial	Nod^+^	Fix^+^	Highly effective
*T. tumens*	1986267	Europe	Perennial	Nod^+^	Fix^-^	Ineffective
*T. tumens*	16758246	Europe	Perennial	Nod^+^	Fix^-^	Ineffective
*T. medium*	21881154	Europe	Perennial	Nod^+^	Fix^-^	Ineffective
*T. repens*	037701	Europe	Perennial	Nod^+^	Fix^-^	Ineffective
*T. repens*	036120	Europe	Perennial	Nod^+^	Fix^-^	Ineffective
*T. pratense*	Russian no 9	Europe	Perennial	Nod^+^	Fix^-^	Ineffective
*T. pratense*	Redquin	Europe	Perennial	Nod^+^	Fix^-^	Ineffective
*T. ambiguum*	Endura	Europe	Perennial	Nod^+^	Fix^-^	Ineffective
*T. canescens*	PL4188661999	Europe	Perennial	Nod^+^	Fix^-^	Ineffective
*T. fragiferum*	C1212	Europe	Perennial	Nod^-^		No nodulation
*T. polymorphum*	87102	South America	Perennial	Nod^+^	Fix^-^	Ineffective
*T. longipes*	A2436817	North America	Perennial	Nod^-^		No nodulation
*T. subterraneum*	York	Europe	Annual	Nod^+^	Fix^-^	Ineffective
*T. glanduliferum*	CP187182	Europe	Annual	Nod^+^	Fix^-^	Ineffective
*T. mulinerve*	87259	Africa	Annual	Nod^-^		No nodulation
*T. tridentatum*	CQ1263	North America	Annual	Nod^+^	Fix^-^	Ineffective

## Genome sequencing and annotation

### Genome project history

This organism was selected for sequencing on the basis of its environmental and agricultural relevance to issues in global carbon cycling, alternative energy production, and biogeochemical importance, and is part of the Community Sequencing Program at the U.S. Department of Energy, Joint Genome Institute (JGI) for projects of relevance to agency missions. The genome project is deposited in the Genomes OnLine Database [28] and a finished genome sequence in IMG/GEBA. Sequencing, finishing and annotation were performed by the JGI. A summary of the project information is shown in Table 3.

**Table 3:** Genome sequencing project information for *Rhizobium leguminosarum* bv. *trifolii* strain WSM1689.

**MIGS ID**	**Property**	**Term**
MIGS-31	Finishing quality	Finished
MIGS-28	Libraries used	Illumina GAii shotgun and paired end 454 libraries
MIGS-29	Sequencing platforms	Illumina GAii and 454 GS FLX Titanium technologies
MIGS-31.2	Sequencing coverage	8.3x 454, 774.6x Illumina
MIGS-30	Assemblers	VELVET, version 1.1.05; Newbler, version 2.6; phrap, version SPS - 4.24
MIGS-32	Gene calling methods	Prodigal 1.4, GenePRIMP
	Genbank ID	Not yet available
	Genbank Date of Release	Not yet released
	GOLD ID	Gi06499
	NCBI project ID	62289
	Database: IMG-GEBA	2510065019
	Project relevance	Symbiotic nitrogen fixation, agriculture

### Growth conditions and DNA isolation

*Rhizobium leguminosarum* bv. *trifolii* strain WSM1689 was grown to mid logarithmic phase in TY rich medium on a gyratory shaker at 28°C [29]. DNA was isolated from 60 mL of cells using a CTAB (Cetyl trimethyl ammonium bromide) bacterial genomic DNA isolation method [30].

### Genome sequencing and assembly

The genome of *Rhizobium leguminosarum* bv. *trifolii* strain WSM1689 was sequenced at the Joint Genome Institute (JGI) using a combination of Illumina [31] and 454 technologies [32]. An Illumina GAii shotgun library which generated 73,565,648 reads totaling 5,591 Mbp, and a paired end 454 library with an average insert size of 12 Kbp which generated 376,185 reads totaling 93.4 Mbp of 454 data were generated for this genome. All general aspects of library construction and sequencing performed at the JGI can be found at [30]. The initial draft assembly contained 100 contigs in 4 scaffolds. The 454 paired end data was assembled with Newbler, version 2.6. The Newbler consensus sequences were computationally shredded into 2 Kbp overlapping fake reads (shreds). Illumina sequencing data was assembled with VELVET, version 1.1.05 [33], and the consensus sequence computationally shredded into 1.5 Kbp overlapping fake reads (shreds). We integrated the 454 Newbler consensus shreds, the Illumina VELVET consensus shreds and the read pairs in the 454 paired end library using parallel phrap, version SPS - 4.24 (High Performance Software, LLC). The software Consed [34-36] was used in the following finishing process. Illumina data was used to correct potential base errors and increase consensus quality using the software Polisher developed at JGI (Alla Lapidus, unpublished). Possible mis-assemblies were corrected using gapResolution (Cliff Han, unpublished), Dupfinisher [37], or sequencing cloned bridging PCR fragments with subcloning. Gaps between contigs were closed by editing in Consed, by PCR and by Bubble PCR (J-F Cheng, unpublished) primer walks. A total of 93 additional reactions were necessary to close gaps and to raise the quality of the finished sequence. The total genome size is 6.9 Mbp and the final assembly is based on 57.3 Mbp of 454 draft data which provides an average 8.3× coverage of the genome and 5,345 Mbp of Illumina draft data which provides an average 774.6× coverage of the genome.

### Genome annotation

Genes were identified using Prodigal [38] as part of the DOE-JGI genome annotation pipeline, followed by a round of manual curation using the JGI GenePRIMP pipeline [39]. The predicted CDSs were translated and used to search the National Center for Biotechnology Information (NCBI) nonredundant database, UniProt, TIGRFam, Pfam, PRIAM, KEGG, COG, and InterPro databases. These data sources were combined to assert a product description for each predicted protein. Non-coding genes and miscellaneous features were predicted using tRNAscan-SE [40], RNAMMer [41], Rfam [42], TMHMM [43], and SignalP [44]. Additional gene prediction analyses and functional annotation were performed within the Integrated Microbial Genomes (IMG-ER) platform [45,46].

## Genome properties

The genome is 6,903,379 nucleotides with 60.94% GC content (Table 4 and Figures 3a,3b,3c,3d,3e and Figure 3f), and comprised of 6 replicons. From a total of 6,798 genes, 6,709 were protein encoding and 89 RNA only encoding genes. Within the genome, 206 pseudogenes were also identified. The majority of genes (79.52%) were assigned a putative function whilst the remaining genes were annotated as hypothetical. The distribution of genes into COGs functional categories is presented in Table 5.

**Table 4:** Genome Statistics for *Rhizobium leguminosarum* bv. *trifolii* strain WSM1689.

**Attribute**	**Value**	**% of Total**
Genome size (bp)	6,903,379	100.00
DNA coding region (bp)	6,004,795	86.98
DNA G+C content (bp)	4,206,909	60.94
Number of replicons	6	
Total genes	6,798	100.00
RNA genes	89	1.31
Protein-coding genes	6,709	98.69
Genes with function prediction	5,406	79.52
Genes assigned to COGs	5,400	79.44
Genes assigned Pfam domains	5,618	82.64
Genes with signal peptides	591	8.69
Genes coding transmembrane proteins	1,524	22.42
CRISPR repeats	0	

**Figure 3a:**
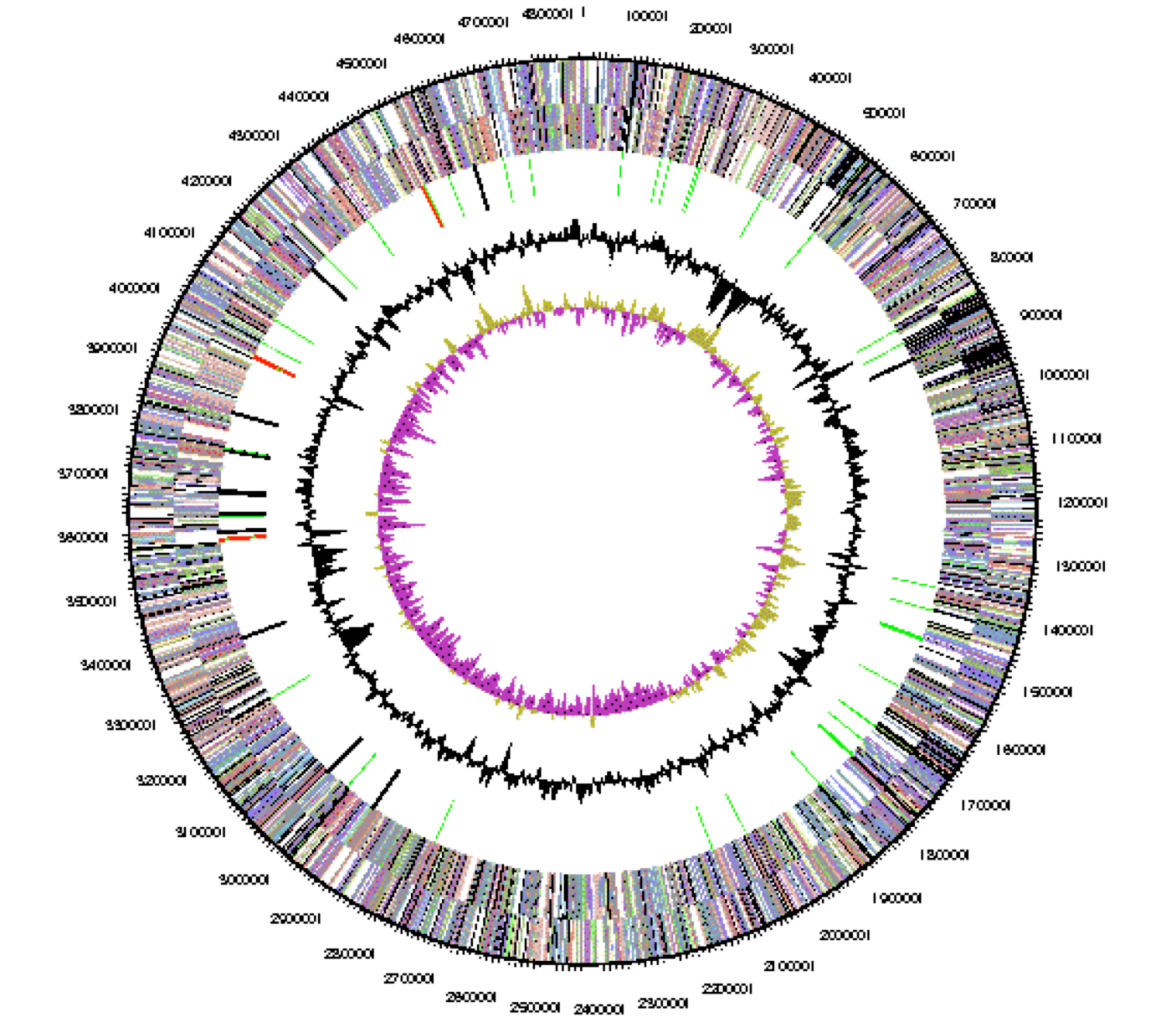
Graphical circular map of Replicon WSM1689_Rleg3_Contig1814.1 of the *Rhizobium leguminosarum* bv. *trifolii* strain WSM1689 genome. From outside to the center: Genes on forward strand (color by COG categories as denoted by the IMG platform), Genes on reverse strand (color by COG categories), RNA genes (tRNAs green, sRNAs red, other RNAs black), GC content, GC skew.

**Figure 3b:**
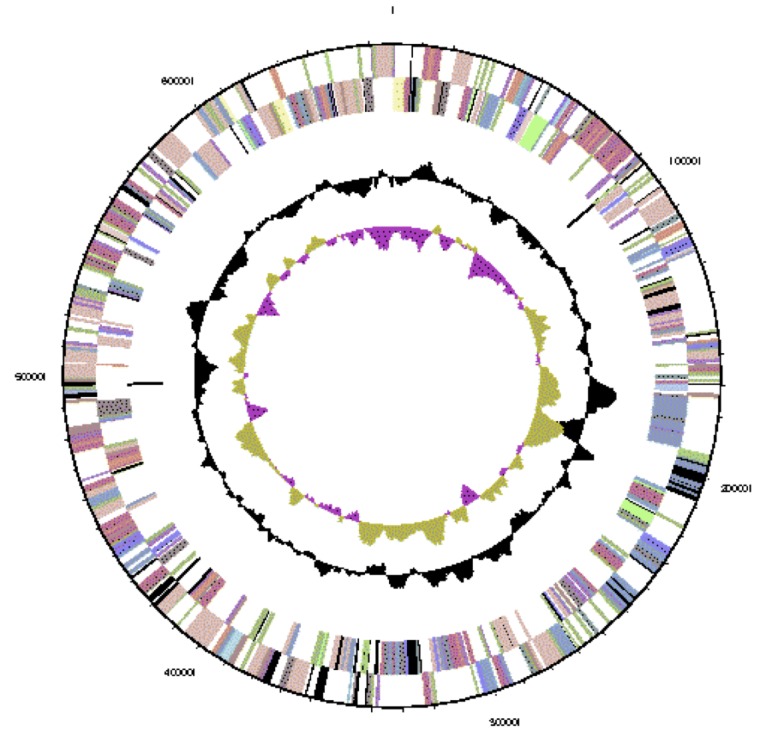
Graphical circular map of replicon WSM1689_Rleg3_Contig1813.2 of the *Rhizobium leguminosarum* bv. *trifolii* strain WSM1689 genome. From outside to the center: Genes on forward strand (color by COG categories as denoted by the IMG platform), Genes on reverse strand (color by COG categories), RNA genes (tRNAs green, sRNAs red, other RNAs black), GC content, GC skew.

**Figure 3c:**
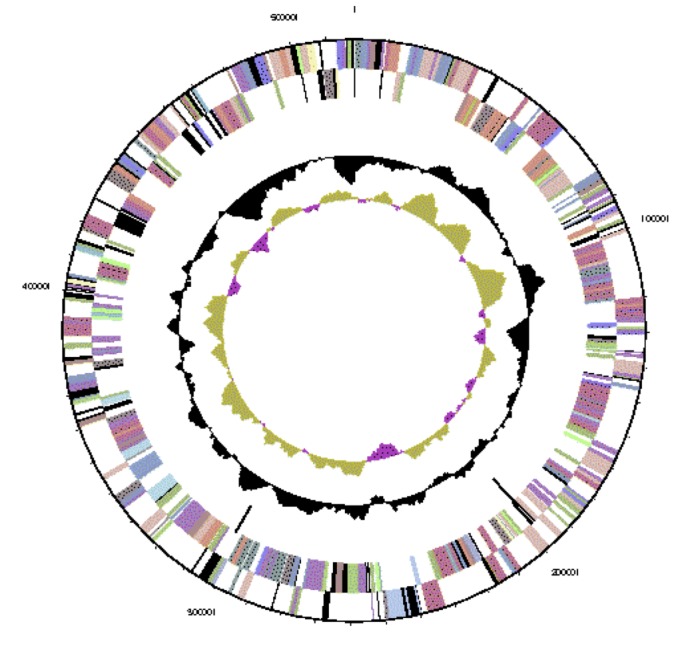
Graphical circular map of replicon WSM1689_Rleg3_Contig1812.3 of the *Rhizobium leguminosarum* bv. *trifolii* strain WSM1689 genome. From outside to the center: Genes on forward strand (color by COG categories as denoted by the IMG platform), Genes on reverse strand (color by COG categories), RNA genes (tRNAs green, sRNAs red, other RNAs black), GC content, GC skew.

**Figure 3d:**
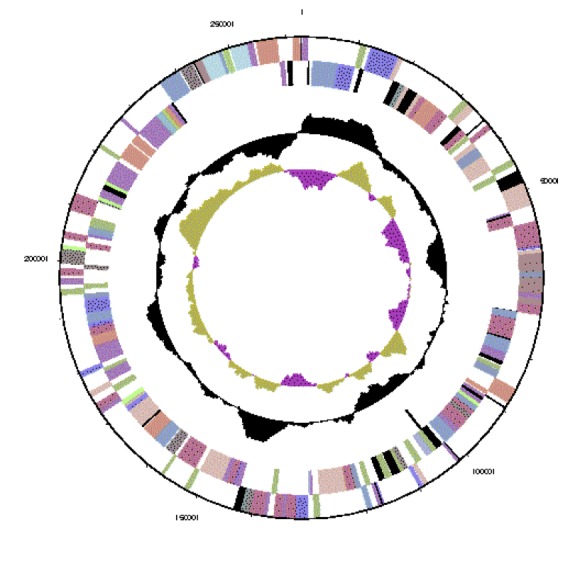
Graphical circular map of replicon WSM1689_Rleg3_Contig1810.5 of the *Rhizobium leguminosarum* bv. *trifolii* strain WSM1689 genome. From outside to the center: Genes on forward strand (color by COG categories as denoted by the IMG platform), Genes on reverse strand (color by COG categories), RNA genes (tRNAs green, sRNAs red, other RNAs black), GC content, GC skew.

**Figure 3e:**
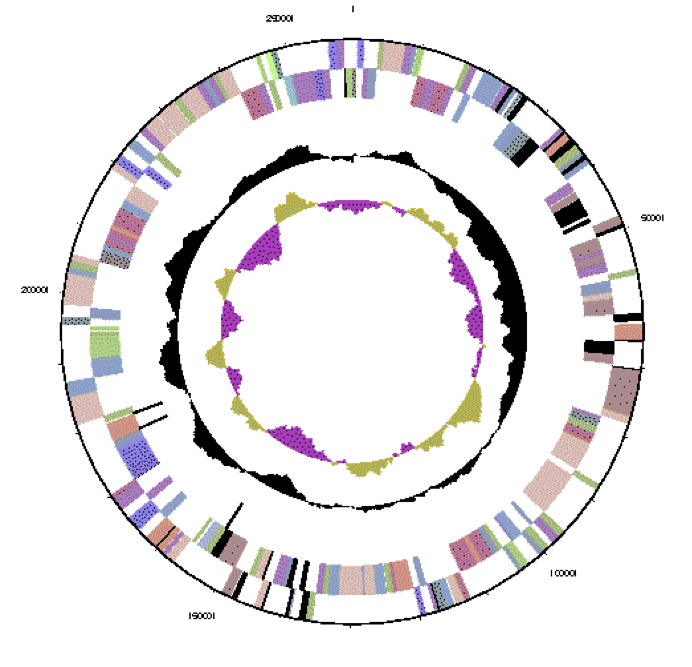
Graphical circular map of replicon WSM1689_Rleg3_Contig1811.4 of the *Rhizobium leguminosarum* bv. *trifolii* strain WSM1689 genome. From outside to the center: Genes on forward strand (color by COG categories as denoted by the IMG platform), Genes on reverse strand (color by COG categories), RNA genes (tRNAs green, sRNAs red, other RNAs black), GC content, GC skew.

**Figure 3f:**
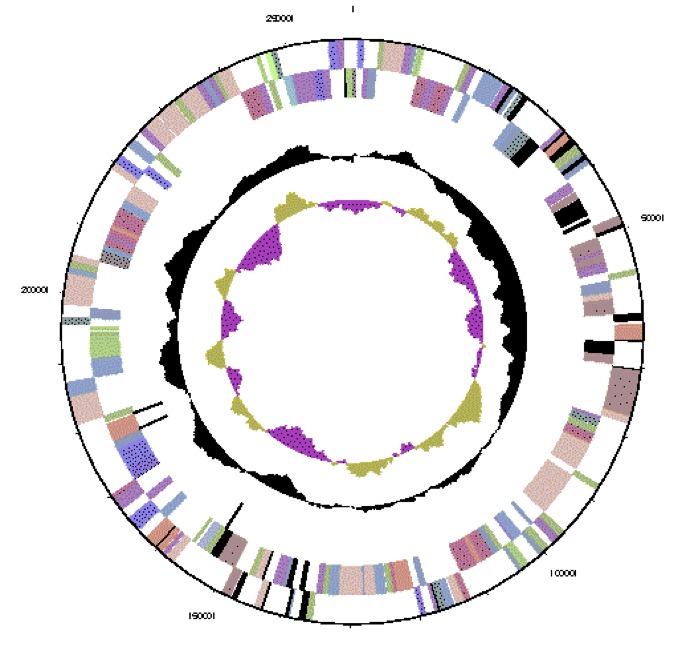
Graphical circular map of replicon WSM1689_Rleg3_Contig1809.6 of the *Rhizobium leguminosarum* bv. *trifolii* strain WSM1689 genome. From outside to the center: Genes on forward strand (color by COG categories as denoted by the IMG platform), Genes on reverse strand (color by COG categories), RNA genes (tRNAs green, sRNAs red, other RNAs black), GC content, GC skew.

**Table 5:** Number of protein coding genes of *Rhizobium leguminosarum* bv. *trifolii* strain WSM1689 associated with the general COG functional categories.

**Code**	**Value**	**%age**	**COG Category**
J	205	3.40	Translation, ribosomal structure and biogenesis
A	0	0.00	RNA processing and modification
K	581	9.62	Transcription
L	153	2.53	Replication, recombination and repair
B	2	0.03	Chromatin structure and dynamics
D	39	0.65	Cell cycle control, mitosis and meiosis
Y	0	0.00	Nuclear structure
V	66	1.09	Defense mechanisms
T	311	5.15	Signal transduction mechanisms
M	329	5.45	Cell wall/membrane biogenesis
N	81	1.34	Cell motility
Z	0	0.00	Cytoskeleton
W	0	0.00	Extracellular structures
U	82	1.36	Intracellular trafficking and secretion
O	187	3.10	Posttranslational modification, protein turnover, chaperones
C	311	5.15	Energy production conversion
G	683	11.31	Carbohydrate transport and metabolism
E	629	10.42	Amino acid transport metabolism
F	105	1.74	Nucleotide transport and metabolism
H	192	3.18	Coenzyme transport and metabolism
I	222	3.68	Lipid transport and metabolism
P	297	4.92	Inorganic ion transport and metabolism
Q	147	2.43	Secondary metabolite biosynthesis, transport and catabolism
R	795	13.17	General function prediction only
S	620	10.27	Function unknown
-	1,398	20.56	Not in COGS
-	6,037	-	Total
